# p53 mutant-type in human prostate cancer cells determines the sensitivity to phenethyl isothiocyanate induced growth inhibition

**DOI:** 10.1186/s13046-019-1267-z

**Published:** 2019-07-15

**Authors:** Monika Aggarwal, Rahul Saxena, Nasir Asif, Elizabeth Sinclair, Judy Tan, Idalia Cruz, Deborah Berry, Bhaskar Kallakury, Quynhchi Pham, Thomas T. Y. Wang, Fung-Lung Chung

**Affiliations:** 1 Department of Oncology, Lombardi Comprehensive Cancer Center, Georgetown University, Washington DC, 20007 USA; 2 Department of Biochemistry and Molecular and Cellular Biology, Georgetown University, Washington DC, 20007 USA; 3 Diet, Genomics and Immunology Laboratory, United States Department of Agriculture, Beltsville, MD 20705 USA

**Keywords:** Tumor suppressor protein, p53 mutation, Reactivation, Phenethyl isothiocyanate, Prostate cancer, Chemoprevention

## Abstract

**Background:**

We reported previously that phenethyl isothiocyanate (PEITC), a dietary compound, can reactivate p53^R175H^ mutant in vitro and in SK-BR-3 (p53^R175H^) breast xenograft model resulting in tumor inhibition. Because of the diversity of human cancers with p53 mutations, these findings raise important questions whether this mechanism operates in different cancer types with same or different p53 mutations. In this study, we investigated whether PEITC recuses mutant p53 in prostate cancer cells harboring different types of p53 mutants, structural and contact, in vitro and in vivo.

**Methods:**

Cell proliferation, cell apoptosis and cell cycle arrest assays were performed to examine the effects of PEITC on prostate cancer cell lines with p53 mutation(s), wild-type p53, p53 null or normal prostate cells in vitro. Western blot analysis was used to monitor the expression levels of p53 protein, activation of ATM and upregulation of canonical p53 targets. Immunoprecipitation, subcellular protein fraction and qRT-PCR was performed to determine change in conformation and restoration of transactivation functions/ inhibition of gain-of-function (GOF) activities to p53 mutant(s). Mice xenograft models were established to evaluate the antitumor efficacy of PEITC and PEITC-induced reactivation of p53 mutant(s) in vivo. Immunohistochemistry of xenograft tumor tissues was performed to determine effects of PEITC on expression of Ki67 and mutant p53 in vivo.

**Results:**

We demonstrated that PEITC inhibits the growth of prostate cancer cells with different “hotspot” p53 mutations (structural and contact), however, preferentially towards structural mutants. PEITC inhibits proliferation and induces apoptosis by rescuing mutant p53 in p53^R248W^ contact (VCaP) and p53^R175H^ structural (LAPC-4) mutant cells with differential potency. We further showed that PEITC inhibits the growth of DU145 cells that co-express p53^P223L^ (structural) and p53^V274F^ (contact) mutants by targeting p53^P223L^ mutant selectively, but not p53^V274F^. The mutant p53 restored by PEITC induces apoptosis in DU145 cells by activating canonical p53 targets, delaying cells in G1 phase and phosphorylating ATM. Importantly, PEITC reactivated p53^R175H^ and p53^P223L/V274F^ mutants in LAPC-4 and DU145 prostate xenograft models, respectively, resulting in significant tumor inhibition.

**Conclusion:**

Our studies provide the first evidence that PEITC’s anti-cancer activity is cancer cell type-independent, but p53 mutant-type dependent.

**Electronic supplementary material:**

The online version of this article (10.1186/s13046-019-1267-z) contains supplementary material, which is available to authorized users.

## Background

Studies on dietary compounds that target p53 mutants are rare. These compounds are important as they may potentially lead to the development of practical and effective approaches for gene-targeted cancer prevention and treatment. Isothiocyanates (ITCs) are a family of compounds rich in cruciferous vegetables that have been shown to inhibit tumorigenesis in a variety of animal models [1, 2]. The cancer protective role of ITCs has been supported by numerous epidemiological studies [3]. Several mechanisms have been studied for cancer chemopreventive activities of ITCs, such as induction of phase II enzymes and inhibition of cytochrome p450, induction of oxidative stress and alterations in several signaling pathways, probably mediated by glutathione depletion and / or inhibition of the mitochondria respiratory chain [1–11]. However, the crucial molecular targets of ITC are still not fully understood. As strong electrophiles, the protein modifications by ITCs may constitute the molecular basis for some of their activities [12, 13].

Earlier we reported that phenethyl isothiocyanate (PEITC), abundant in watercress, selectively depletes mutant p53, but not wild-type (WT) p53, in a variety of human cancer cell lines [14]. Recently, we uncovered a novel mechanism that PEITC can target p53^R175H^ mutant in breast cancer SK-BR-3 cells in vitro, and, inhibits its growth in a xenograft mouse model [15]. We demonstrated that PEITC induces apoptosis in these cells by restoring the WT p53 functions to p53^R175H^ mutant and by activating the canonical p53 targets, resulting in delay in cell cycle progression. Furthermore, PEITC renders p53^R175H^ mutant sensitive to degradation by proteasome degradation pathway. These results suggest that depletion of mutant p53 by PEITC via restoration of its WT form may be responsible for its activity. This study for the first time unraveled that a dietary compound can restore the WT functions to mutant p53. Given the diversity of human cancers with p53 mutations, types of p53 mutants (structural and contact) [16] and the pivotal role of p53 in cancer development, our findings raise important questions whether reactivation of mutant p53 by PEITC applies to other types of cancer cells expressing p53^R175H^ mutant or cells harboring different p53 mutants. To address these questions, we investigated this mechanism in prostate cancer cells expressing different p53 mutations, including p53^R175H^ mutant. Prostate cancer is the leading cause of cancer-related deaths in American men, after lung cancer, and has significantly high frequency of p53 gene mutation (~ 30–70%) [17, 18]. Studies showed that mutations in the p53 gene occur in the initiation, progression and metastasis stages of this disease [17–20], thus, playing a critical role throughout prostate cancer development. Despite this, therapeutic options targeting p53 mutant in prostate cancer patients are lacking.

## Materials and methods

### Cell lines

DU145 and PC-3 cells were obtained from Tissue Culture Source Resource, Georgetown University, Washington, DC. The cells were cultured in RPMI 1640 with 10 and 7% fetal bovine serum (FBS), respectively. Normal prostate cells RWPE2 and VCaP cells were purchased from ATCC. The RWPE2 cells were cultured in Keratinocyte serum free medium containing 0.05 mg/ml bovine pituitary extract and 5 ng/ml human recombinant epidermal growth factor and VCaP cells were cultured in ATCC-formulated Dulbecco’s modified Eagle’s medium with 15% FBS. The cell line LAPC-4 was a gift from Dr. Charles L. Sawyers. The LAPC-4 cells were cultured in Iscove’s Modified Dulbecco Media with 10% FBS. The LNCaP cells were provided by Dr. Thomas T.Y. Wang and were cultured in RPMI 1640 with 10% FBS. All the cell lines were negative for mycoplasma.

### Cell proliferation assays

The effect of PEITC on LAPC-4 and VCaP cells proliferation was determined by using the WST-1 assay (Sigma) as described previously [15]. Briefly, PEITC was diluted in DMSO so that 10 μl of diluted stock in a 1 ml aliquot of cells (40,000 cells /ml) yielded a desired concentration of PEITC at 1% DMSO. Cell cultures containing PEITC were plated onto a 96-well microtiter plate at 4000 cells per well in duplicate. As a control, 4000 cells per well were seeded in medium containing 1% DMSO in duplicate. For background subtraction, wells lacking cells but containing medium were used. Plates were incubated at 37 °C for 24 h, followed by the addition of WST-1 reagent for 2 h. OD_450_ was measured using a microplate reader (Bio-Rad). Percent cell proliferation was calculated as the ratio of OD_450_ values obtained for respective cells grown in the presence of PEITC compared with the presence of DMSO. Similar assays were performed to determine the effect of PEITC on proliferation of RWPE2, LNCaP, PC-3, DU145, PC-3 cells transfected with pCMV-Neo-Bam, pCMV-Neo –Bam-WTp53, pCMV-Neo –Bam-p53P223L, pCMV-Neo –Bam-p53V274F, pCMV-Neo –Bam-p53R175H or pCMV-Neo –Bam-p53R248W, DU145 cells transfected with pcDNA3 or HA-p73α-pcDNA3 and LAPC-4 cells transfected with non-specific (NS) siRNA or p53 siRNA. The NS siRNA and p53 siRNA were obtained from Thermo Scientific/ Dharmacom, Lafayette, CO, USA.

### Construction of plasmids

Site-directed mutagenesis technique was used to generate p53^P223L^ and p53^V274F^ mutants. The p53 gene with mutation at P223L was amplified using the forward mutagenic SA9 (5′-CCGTTAGAGGTTGGCTCTGACTCT-3′) and the reverse mutagenic SA10 (5′-AACCTCTAACGGCTCATAGGGCAC-3′) primers. Similarly, for mutation at V274F the forward mutagenic SA11 (5′-GTGCGTTTTTGTGCCTGTCCTG-3′) and the reverse mutagenic SA12 (5′-GGCACAAAAACGCACCTCAAAGTC-3′) primers were used. Further, for both the mutations the forward SA7 (5′-ACCTATCTAGAATGGAGGAGCCGCAGTCA-3′) and reverse SA8 (5′-AACCGGATCCTCAGTCTGAGTCAGGCCC-3′) end primers were used.

Amplicons were sub-cloned into Xba1 and BamH1 sites in the pCMV-Neo-Bam plasmid. The plasmid pCMV-Neo-Bam was a gift from Bert Vogelstein (Addgene plasmid no. 16440) [21]. Correct insertion was confirmed by gel electrophoresis and the mutations were confirmed by DNA sequencing.

### Transfection in cells

The plasmids were transfected using Lipofectamine 2000 following the manufacturer’s protocol (Invitrogen) as described previously [15]. Briefly, cells were plated to 50–60% confluence in 10-cm dishes 24 h before transfection. The plasmid (4 μg) was mixed with 25 μl of Lipofectamine 2000 in 1 ml of Opti-MEM (Invitrogen). The mixture was added to cells that subsequently were incubated for 5 h. After 24 h, transfected cells were treated with PEITC for WST-1 assay or Annexin V staining as described. The transfected cells were maintained in RPMI 1640 with 7% FBS for PC-3 and 10% FBS for DU145 cells, respectively, and 400 μg/ml G418. The plasmid pCMV-Neo-Bam-WTp53, pCMV-Neo –Bam-p53R175H and pCMV-Neo –Bam-p53R248W and HA-p73α-pcDNA3 were gifts from Bert Vogelstein (Addgene plasmid no. 16434, 16436 and 16437) and William Kaelin (Addgene plasmid no. 22102) [21, 22].

The siRNA was transfected using Lipofectamine 2000 as described previously [15]. Briefly, LAPC-4 cells were plated to 50–60% confluence in 10 cm dishes 24 h before transfection. The siRNA (0.430 nmol) was mixed with 43 μl of Lipofectamine 2000 in 1 ml of Opti-MEM (Invitrogen). The mixture was added to the cells that subsequently were incubated for 5 h. After 24 h, a second transfection was performed similarly. Seventy-two hours after the initial transfection, cells were treated with PEITC or DMSO at the indicated concentrations and cell proliferation was measured by using the WST-1 reagent (Sigma) as described previously.

### Annexin V staining

The Annexin V staining was done in accordance with the manufacturer’s instructions (Biolegend). In brief, DU145, LNCaP, PC-3, LAPC-4, VCaP and DU145 cells transfected with pcDNA3 or HA-p73α-pcDNA3 were treated with PEITC as indicated or DMSO as a control for 24 h. Cells were harvested by scraping, washed once with 1 x PBS and resuspended in 0.5 ml Annexin V binding buffer. Cells were collected by centrifugation, 5 μl of the fluorochrome conjugated Annexin V was added in the residual buffer and cells were incubated at RT in the dark for 15 min followed by the addition of 0.5 ml of Annexin V binding buffer and 5 μl of PI staining solution (0.1 μg/ml). Cells were then analyzed by flow cytometry using a BD LSRFORTESSA instrument (BD Biosciences).

### Immunoprecipitation

DU145, PC-3, LAPC-4 and VCaP cells were treated with indicated concentration of PEITC or DMSO as a control for 5 h. Cells were harvested, washed once with 1 x PBS and cell pellets were suspended in lysis buffer (20 mM Tris-Cl (pH 8.0), 137 mM sodium chloride, 10% glycerol, 1% NP-40, 2 mM EDTA) containing protease inhibitors cocktail (Roche Molecular Biochemicals). The cells were incubated on ice for 30 min. The cell suspension was centrifuged at 18,500 × g for 10 min at 4 °C, and supernatant was collected. The supernatants were diluted in lysis buffer and 200 μg of the lysate was gently tumbled at 4 °C for 1 h with protein G–agarose beads (Roche). The lysates obtained after pre-clearing were then gently tumbled at 4 °C for 2 h with mouse PAB240 antibody (2 μg, Calbiochem). Protein G–agarose beads were then added to the suspensions and incubation was performed for 2 h at 4 °C. The beads were washed four times with lysis buffer supplemented with protease inhibitors and the immunoprecipitates were eluted by boiling in Laemmli buffer and resolved on 4–12% SDS–PAGE. Immunoprecipitated p53 was detected by Western blotting using general anti-p53 FL393 (Santa Cruz Biotechnology) as a primary antibody. For the secondary antibody, peroxidase-labeled anti-mouse IgG (1:2000, GE healthcare) was used. The blot was developed using the ECL Prime Western Blot Detection Kit according to the manufacturer’s protocol (Amersham). As a control, the input lysate blot was probed with a general anti-p53 (DO-1) antibody (1: 1000, Santa Cruz Biotechnology). Blot was stripped and reprobed with anti-GAPDH antibody (1:5000, Novus Biologicals).

### Lysate preparation and western blot analysis

DU145, VCaP, LAPC-4, PC-3 cells transfected with pCMV-Neo-Bam, pCMV-Neo-Bam-WTp53, pCMV-Neo-Bam-p53P223L, pCMV-Neo-Bam-p53V274F, pCMV-Neo –Bam-p53R175H or pCMV-Neo –Bam-p53R248W and DU145 cells transfected with pcDNA3 or HA-p73α-pcDNA3 were either untreated or treated with PEITC or DMSO for 4 h. Cells were then harvested by centrifugation at 1600 × g for 10 min at 4 °C, washed once with PBS, resuspended in lysis buffer (as described above) containing a protease inhibitor cocktail (Roche Molecular Biochemicals) and were then incubated on ice for 30 min. The lysates were centrifuged at 18,500 × g for 10 min at 4 °C. Then 30–50 μg of the lysates were loaded on 4–12% SDS-PAGE. Protein was transferred onto a PVDF membrane, and the blots were developed using the ECL Prime Western Blot Detection Kit according to the manufacturer’s protocol (Amersham). The antibodies for p53 (DO-1) and p73 were purchased from Santa Cruz Biotechnology and GAPDH antibody was from Novus Biologicals.

For detecting phsophorylation of ATM, 200 μg of the lysate was loaded on 4–12% SDS-PAGE. Following electrophoresis, protein was transferred onto a PVDF membrane, and blot was probed with anti-pATM Ser1981 antibody (1:500) (Santa Cruz Biotechnology). For the secondary antibody, peroxidase-labeled anti-mouse IgG (1:1000, GE Healthcare) was used. The blot was developed using the ECL Prime Western Blot Detection Kit following the manufacturer’s protocol (Amersham). As a control, the blot was probed with anti- GAPDH antibody (1: 5000, Novus Biologicals).

### Chromatin fractionation

DU145 cells were treated with indicated concentrations of PEITC or DMSO as a control for 4 h. Cells were harvested by centrifugation at 500 × g for 5 min and pellets were washed once with ice-cold PBS. Cells were transferred to 1.5 ml microcentrifuge tubes followed by centrifugation at 500 × g for 2 min. Pellets were stored at − 80 °C prior to chromatin fractionation. Nuclear soluble and chromatin-bound fractions were then prepared following the manufacturer’s instruction (Subcellular protein fractionation kit, Thermo Scientific). Ten μg of protein from the soluble nuclear extract and the chromatin-bound nuclear extract for the samples from DMSO- or PEITC-treated cells were resolved on 4–12% SDS-PAGE and transferred to PVDF membranes. Blots were probed with p53 (DO-1) antibody (1:1000, Santa Cruz Biotechnology). Histone H3 and TopoIIB served as markers for the chromatin and soluble nuclear fractions, respectively. The markers were detected using rabbit anti-Histone H3 polyclonal (Thermo Scientific) and mouse anti-TopoIIB monoclonal (Santa Cruz Biotechnology) antibodies, respectively.

### RNA extraction and quantitative real time polymerase chain reaction (qRT-PCR)

DU145, LNCaP, PC-3, LAPC-4, VCaP, PC-3 cells transfected with pCMV-Neo-Bam, pCMV-Neo-Bam-WT p53, pCMV-Neo-Bam-p53P223L, pCMV-Neo-Bam-p53V274F, pCMV-Neo –Bam-p53R175H or pCMV-Neo –Bam-p53R248W were treated with 8 μM PEITC or DMSO as a control for 4 h and RNA was extracted using a Qiagen RNeasy Kit. cDNA was then synthesized by using High Capacity RNA to cDNA kit (Applied Biosystems, Invitrogen) and the gene expression level was measured by qRT-PCR using TaqMan gene expression assays (Applied Biosystems, Invitrogen). The gene expression level was normalized with GAPDH, and the average is presented with standard deviation from triplicates of repeated experiments.

RNA was extracted from the LAPC-4 and DU145 xenograft tissues by using RNA extraction procedure as described above for the cells. The gene expression levels were normalized with GAPDH. Fold changes in the gene expression levels were calculated for each tumor from the PEITC-treated group relative to the tumors from the control group and the average is presented with standard deviation.

### Cell cycle analysis

DU145, LAPC-4, LNCaP and PC-3 cells were treated with PEITC or DMSO as a control for 24 h. Cells were then prepared for flow cytometric analysis. Briefly, cells were washed with PBS free of Ca^2+^ and Mg^2+^, trypsinized for 5 min, and harvested by centrifugation at 190 x g for 3 min at 4 °C. Cells were washed once with PBS and pellets resuspended in 1 ml of 70% ethanol and stored at − 20 °C overnight. Cells were harvested by centrifugation at 420 x g for 10 min. The cell pellets were washed once with 1 ml cold PBS and resuspended in 1 ml freshly prepared PI staining solution (PBS with 0.1% Triton X-100, 0.05 μg/ml propidium iodide, 0.1 mg/ml RNase (Sigma)). The cell suspension was incubated at room temperature for 30 min the in dark followed by incubation for 30 min at 4 °C. The samples were run on a Becton Dickinson FACS sort and the data was analyzed using Mod Fit program (Verity Software House).

### Mouse xenograft models

All in vivo studies and tumor harvest were performed as described previously, except that in this study male athymic nu/nu Balb/c mice (CAnN.Cg-Foxn1nu/Crl, 4–6 weeks old) were used and the mice were injected with exponentially grown 2 × 10^6^ LAPC-4 or DU145 cells (suspended in 50 μl of Matrigel) in their left and right flanks [15].

### Histopathological analysis and immunohistochemistry of the LAPC-4 and DU145 tumor xenografts

The tissues were confirmed as tumors by histopathological examination and immunohistochemistry was performed at Histopathology and Tissue Shared Resources, Georgetown University following standard protocols. Briefly, tissues were sectioned at 5 μm, de-paraffinized with xylenes and rehydrated through a graded alcohol series. Heat induced epitope retrieval (HIER) was performed by immersing the tissue sections at 98 °C for 20 min in 10 mM citrate buffer (pH 6.0) with 0.05% Tween. Immunohistochemical staining was performed using a horseradish peroxidase labeled polymer from Dako (K4001, K4003) according to the manufacturer’s instructions. Briefly, slides were treated with 3% hydrogen peroxide and 10% normal goat serum for 10 min each, and exposed to primary antibodies p53 (DO-7) (1:800, Dako) for 1 h at RT and Ki-67(1:15, Novus Biologicals) overnight at 4 °C. Slides were exposed to the appropriate HRP labeled polymer for 30 min and DAB chromagen (Dako) for 5 min. Slides were counterstained with Hematoxylin (Fisher, Harris Modified Hematoxylin), blued in 1% ammonium hydroxide, dehydrated, and mounted with Acrymount. Consecutive sections with the primary antibody omitted were used as negative controls. The sections were examined under an Olympus BX61 microscope at × 200 magnification. Representative images were captured from the entire tumor section using a DP70 camera and DP70 software, and images were analyzed using Image J software. For each antibody ten pictures were taken from different areas on slide to count the number of positively stained cells.

### Western blot and qRT-PCR analysis of the tumor xenografts

For western blot analysis, the DU145 tumor xenograft lysate from each group was prepared by homogenizing the tissue in 20 w/v of lysis buffer (Pierce). 25 μg of the lysates were loaded on 4–12% SDS-PAGE, transferred onto a PVDF membrane, and the blot was probed with p53 (DO-1), p21 or BAX antibodies as described previously. To perform qRT-PCR assay with the LAPC-4 or DU145 tumor xenograft tissues, RNA was extracted from tumor tissues using Qiagen RNeasy Kit and gene expression level was measured as described previously [15].

### Statistical analysis

Statistical differences in tumor volumes and biological endpoints were evaluated with a two-tailed Student’s *t* test. Differences were considered statistically significant at *p* values of ≤0.05. All statistical tests were two-sided.

## Results

### PEITC affects the growth of prostate cancer cells expressing different hotspot p53 mutants

**Fig. 1:**
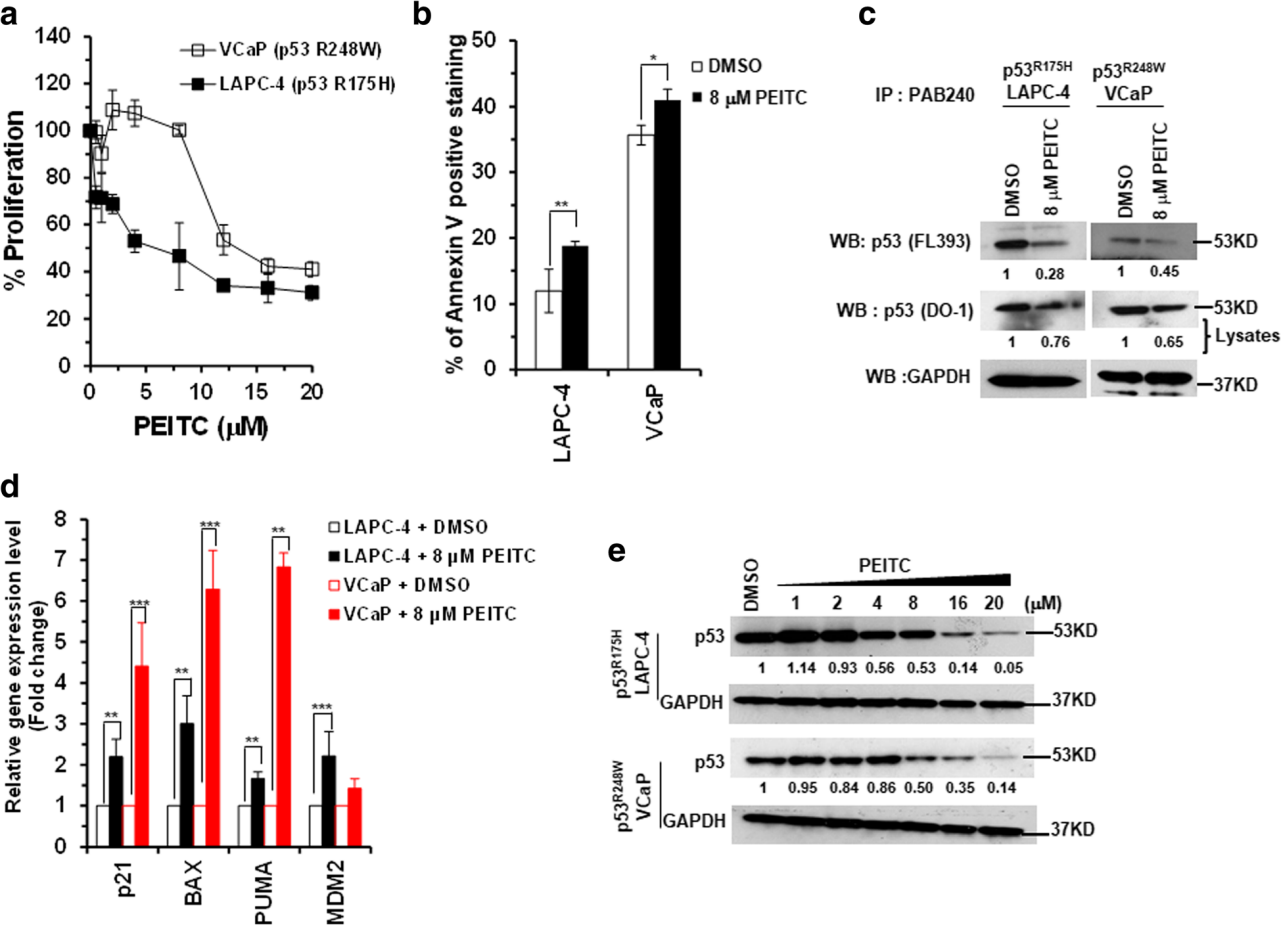
PEITC inhibits cell proliferation, induces apoptosis and reactivates p53 mutants in prostate cancer cell lines. LAPC-4 (p53^R175H^) and VCaP (p53^R248W^) cells were treated with DMSO or PEITC for 24 h. **a** Percent cell proliferation was determined by the WST-1 assay, and **b** Apoptosis was measured by Annexin-V staining by flow cytometry using a BD LSRFORTESSA instrument. (**p ≤ 0.009 and *p ≤ 0.01). **c** Immunoprecipitation of the p53 mutant protein from PEITC treated LAPC-4 and VCaP cell lysates by using p53 mutant-specific antibody PAB240 and detected by a general anti-p53 (FL393) antibody. Input lysates were probed with a general anti-p53 (DO-1) antibody. Blots were stripped and reprobed with anti-GAPDH antibody. **d** qRT-PCR of p53 regulated genes in LAPC-4 and VCaP cells treated with DMSO or 8 μM PEITC for 4 h. (***p ≤ 0.0001 and **p ≤ 0.007). **e** LAPC-4 and VCaP cells were treated with DMSO or the indicated concentration of PEITC for 4 h. The cell lysates were resolved by SDS-PAGE, probed with p53 DO-1 antibody and reprobed with anti-GAPDH antibody

To determine if PEITC inhibits the growth of prostate cancer cells expressing different hotspot p53 mutants and restores transactivation functions, we treated human prostate LAPC-4 (p53^R175H^) (structural mutant) and VCaP (p53^R248W^) (contact mutant) cells, that are homozygous p53 mutant, with PEITC. PEITC inhibited the proliferation of LAPC-4 (p53^R175H^) and VCaP (p53^R248W^) cells (IC_50_ 4 μM and 12 μM, respectively) to differential extent and induced apoptosis (Fig. 1a and b and Additional file 1: Figure S1).

Because PEITC induced apoptosis in LAPC-4 and VCaP cells harboring different p53 mutants, we reasoned that it may do so by restoring WT p53 functions. Therefore, we examined the effects of PEITC on the conformation of p53 mutants by immunoprecipitation using a mutant p53-specific PAB240 antibody. The western blot indicated a significant decrease in PAB240 immunoreactivity in both p53^R175H^ and p53^R248W^ cell lysates (Fig. 1c), thus, demonstrating that PEITC could induce a conformational change in different p53 mutant proteins. Strikingly, consistent with the conformation change, treatment with PEITC enhanced the expression of canonical p53 targets, specifically p21, BAX, PUMA and MDM2 in both LAPC-4 and VCaP cells (Fig. 1d). These results provide strong evidence that PEITC can inhibit the growth of prostate cancer cells expressing different “hotspot” p53 mutants, by reactivating mutant p53, however, with differential potency depending upon the type of mutation.

Next we determined whether reactivation of mutant p53 also affects its expression levels. Previous studies have shown that compounds that reactivate mutant p53 also induce its partial depletion [23]. We demonstrated that PEITC treatment induces the restoration of p53^R175H^ mutant in SK-BR-3 breast cancer cells and the restored p53^R175H^ mutant is sensitive to degradation by proteasome degradation and autophagy pathways [15]. Therefore, to assess the effects of PEITC on p53 mutant protein levels in prostate cancer cells, p53^R175H^ LAPC-4 and p53^R248W^ VCaP cells were treated with PEITC for 4 h. PEITC induced a significant reduction in the expression levels of p53^R175H^ and p53^R248W^ mutants in LAPC-4 and VCaP cells, respectively, demonstrating that it affects the stability of different “hotspot” p53 mutants, with varied potency, in prostate cancer cells (Fig. 1e).

### Effects of PEITC on p53^R175H^ mutant and LAPC-4 xenograft tumor growth in vivo

**Fig. 2:**
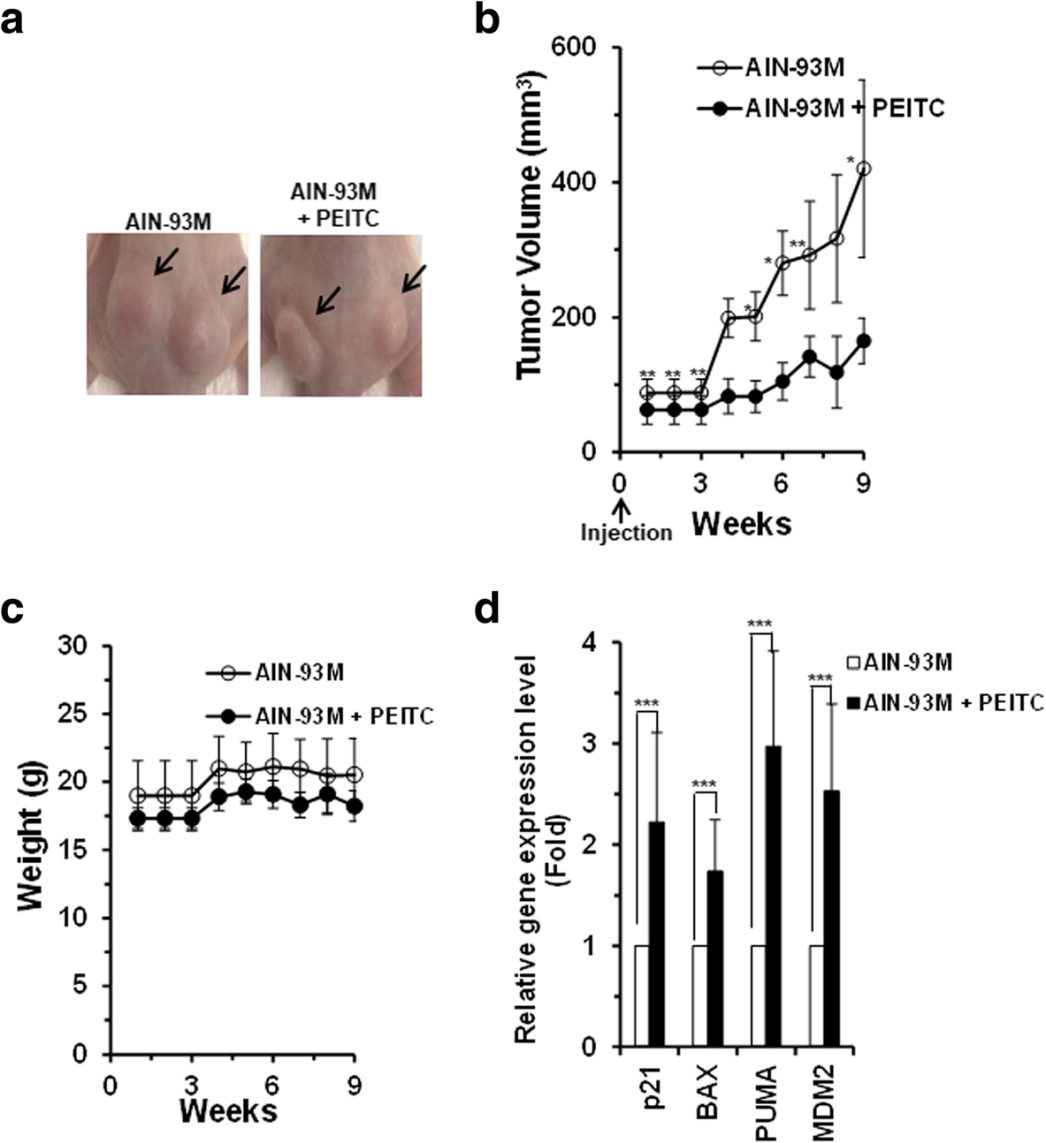
PEITC reactivates p53^R175H^ mutant in LAPC-4 xenograft tumor and inhibits its growth. **a** Representative images of LAPC-4 prostate xenograft tumors in control (Left Panel) and PEITC-treated (Right Panel) animal groups. **b** Tumors volumes in animals. (**p ≤ 0.009 and *p ≤ 0.02) (*n* = 6). **c** Animal weights (g) were measured weekly. **d** qRT-PCR (*n* = 5) of p53 regulated genes in PEITC and control animal groups. (***p ≤ 0.0000)

Previously, we have demonstrated that PEITC reactivated p53^R175H^ mutant in SK-BR-3 breast cancer cells and inhibited xenograft tumor growth in vivo [15]. To determine if PEITC inhibits the growth of other types of cancer cells expressing p53^R175H^ mutant in vivo, we evaluated its ability to inhibit tumor growth in the LAPC-4 prostate xenograft mouse model. Briefly, male athymic nu/nu Balb/c mice (CAnN.Cg-Foxn1nu/Crl, 4–6-week old) was fed a PEITC diet (5 μmol/g AIN-93 M diet) a week before the injection of 2 × 10^6^ LAPC-4 cells into their left and right flanks (“cancer chemoprevention” settings). The animals in the control group were given an AIN-93 M diet. PEITC concentration was chosen based on the earlier work with A/J mice [24]. Tumor formation was assessed and tumor volumes were measured during the 9 weeks bioassay period. A statistically significant inhibition of tumor growth (> 50%, **p ≤ .009 and *p ≤ 0.02) was observed in animals that were given PEITC diet as compared to animals on the control diet (Fig. 2a and b). Of note, we detected a statistically significant decrease in tumor volumes of animal fed PEITC (~ 63 mm^3^) compared to control (~ 88 mm^3^) group starting as early as first week after tumor cells injection (*p* < 0.002). No significant difference in the body weights was observed during the bioassay between the groups (Fig. 2c). These results demonstrate that PEITC has anti-tumor activity in LAPC-4 prostate xenograft model.

To assess PEITC-induced p53^R175H^ mutant reactivation in vivo, we evaluated the levels of canonical WT p53 targets between the groups. We detected statistically signification elevation in mRNA levels of p53 regulated genes (p21, BAX, PUMA and MDM2) from animals fed PEITC diet as compared to those on the control diet (Fig. 2d). These results provide in vivo evidence for the PEITC-induced p53^R175H^ mutant reactivation and inhibition of LAPC-4 prostate xenograft growth.

### Effects of PEITC on proliferation and apoptosis in human prostate DU145 cells

**Fig. 3:**
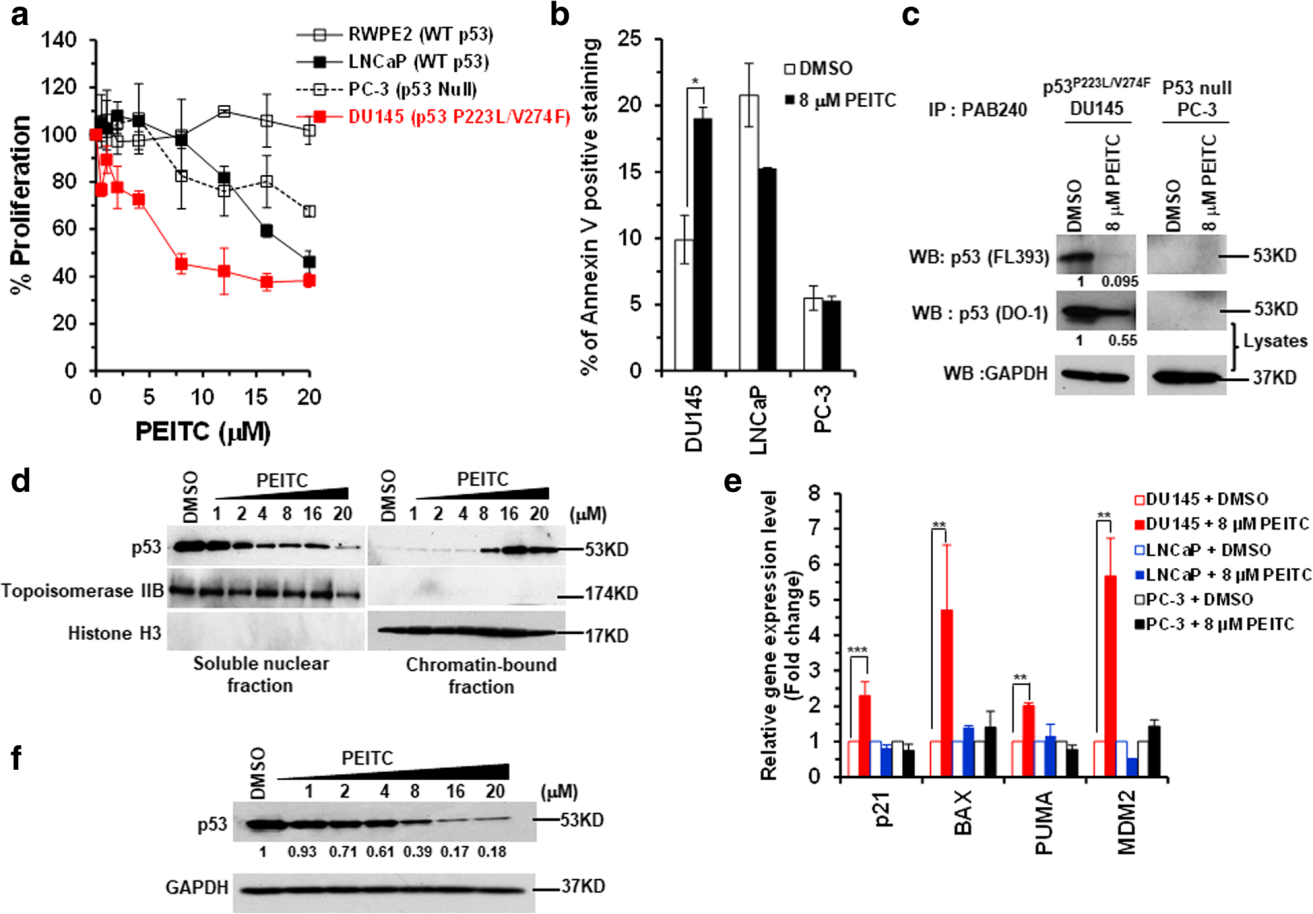
PEITC inhibits cell proliferation, induces apoptosis and reactivates p53^P223L/V274F^ mutant in DU145 cells. Prostate cancer cells with p53 mutations p53^P223L/V274F^ (DU145), WT p53 (LNCaP), p53 null (PC-3) and normal prostate epithelial cells (RWPE2) were treated with DMSO or PEITC for 24 h. **a** Percent cell proliferation was determined by the WST-1 assay, and **b** Apoptosis was measured by Annexin-V staining by flow cytometry using a BD LSRFORTESSA instrument. (*p ≤ 0.02). **c** Immunoprecipitation of the p53 mutant protein from DU145 cell lysates by using p53 mutant-specific antibody PAB240 and detected by a general anti-p53 (FL393) antibody. The PC-3 (p53 null) cells were used as a control for PAB240 antibody. Input lysates were probed with a general anti-p53 (DO-1) antibody. Blots were stripped and reprobed with anti-GAPDH antibody. **d** DU145 cells were treated with PEITC for 4 h and chromatin-bound and soluble nuclear fractions were analyzed by immunoblotting. Histone H3 and Topoisomerase IIB served as markers for the chromatin and soluble nuclear fractions, respectively. **e** qRT-PCR of p53 regulated genes in DU145, LNCaP and PC-3 cells treated with DMSO or 8 μM PEITC for 4 h. (***p ≤ 0.0009 and **p ≤ 0.006). **f** DU145 cells were treated with DMSO or the indicated concentration of PEITC for 4 h. The cell lysate was resolved by SDS-PAGE, probed with p53 DO-1 antibody and re-probed with anti-GAPDH antibody

To further determine how broad the mechanism applies in prostate cancer, we evaluated its ability to inhibit the growth of DU145 cells. We chose to study DU145 cell line that expresses two p53 mutants, p53^P223L^ (structural mutant) and p53^V274F^ (contact mutant), thus presenting a unique opportunity to evaluate the inhibitory effects of PEITC on two different types of p53 mutants in a same genetic background. We compared the effects of PEITC on p53 mutant (DU145) with WT p53 (LNCaP) or p53-null (PC-3) cells and normal prostate epithelial cells (RWPE2, WT p53). PEITC showed the maximum inhibition of cell proliferation for DU145 p53 mutant cells at the lower micromolar concentrations (IC_50_ 8 μM) (Fig. 3a). The p53 mutant DU145 cells exhibited IC_50s_ that were approximately 2.5 folds lower than cells with WT p53 (LNCaP, IC_50_ 20 μM) (Fig. 3a). The reduced cell viability in LNCaP cells at high concentration of PEITC is consistent with a report that PEITC induced apoptosis by increasing p53 expression and activating p53-dependent transcriptional activity, thus, suggesting that PEITC can act in a p53-dependent manner in the WT p53 cells [25]. Consistent with a previous study [26] that PEITC induces apoptosis in p53 deficient cells by activating extracellular signal-regulated kinase (ERK1/2), we also detected mild inhibition of cell proliferation in p53 null PC-3 cells (Fig. 3a). No significant inhibition of cell proliferation was observed for the normal prostate epithelial cell line treated with PEITC (Fig. 3a). DU145 cells treated with 8 μM PEITC displayed an approximate twofold increase in the number of the Annexin-V stained cells (Fig. 3b and Additional file 2: Figure S2), but no significant induction of apoptosis was detected with LNCaP (WT p53) or PC-3 (p53 null) cells (Fig. 3b and Additional file 2: Figure S2). Collectively, these results demonstrate that prostate cancer cells harboring p53 mutant were the most sensitive to the growth inhibition induced by PEITC.

### PEITC induces conformational changes, restores DNA binding and transactivational functions to p53 mutant protein

We examined whether PEITC restores the conformation of p53^P223L/V274F^ mutant by immunoprecipitation using a mutant p53-specific PAB240 antibody. The western blot indicated a significant decrease in PAB240 immunoreactivity in the lysates of DU145 cells treated with PEITC (Fig. 3c). Cell lysates from p53 null PC-3 cells were used as a control for the specificity of PAB240 for mutant p53 protein. These results demonstrate that PEITC induced conformational change in p53 mutant protein.

Because DNA binding is critical for p53 tumor suppressor function, we examined whether PEITC induced conformational change could enrich p53 mutant protein in the chromatin fractions. Western blot analysis of chromatin-bound fractions prepared from DU145 cells treated with PEITC showed a dose-dependent increase in p53 mutant bound to the chromatin (Fig. 3d).

To determine the restoration of the transactivation functions to p53 mutant, we evaluated the mRNA levels of p53 regulated genes (p21, BAX, MDM2 and PUMA) in the PEITC treated DU145 p53^P223L/V274F^ cells. As controls mRNA levels of canonical p53 targets were evaluated after PEITC treatment in LNCaP and PC-3 cells. PEITC (8 μM) significantly enhanced the expression of the WT p53 canonical targets in p53 mutant DU145 cells (Fig. 3e). No significant change in the expression levels was observed in LNCaP and PC-3 cells treated with PEITC suggesting that the induction of p53 targets by PEITC was p53 mediated (Fig. 3e). These results support that PEITC induces conformation changes and restores transactivation functions to p53 mutant in DU145 cells.

To assess the effects of PEITC on p53 mutant protein levels in DU145 cells, cells were treated with PEITC for 4 h. Western blot analysis revealed a significant decrease in p53 mutant protein levels in DU145 cells at PEITC concentrations as low as 8 μM, demonstrating that PEITC treatment destabilizes p53 mutant protein (Fig. 3f).

### Inhibition of p53 mutant cell proliferation and restoration of the transactivational functions by PEITC is p53 mutant-dependent in prostate cancer cells

**Fig. 4:**
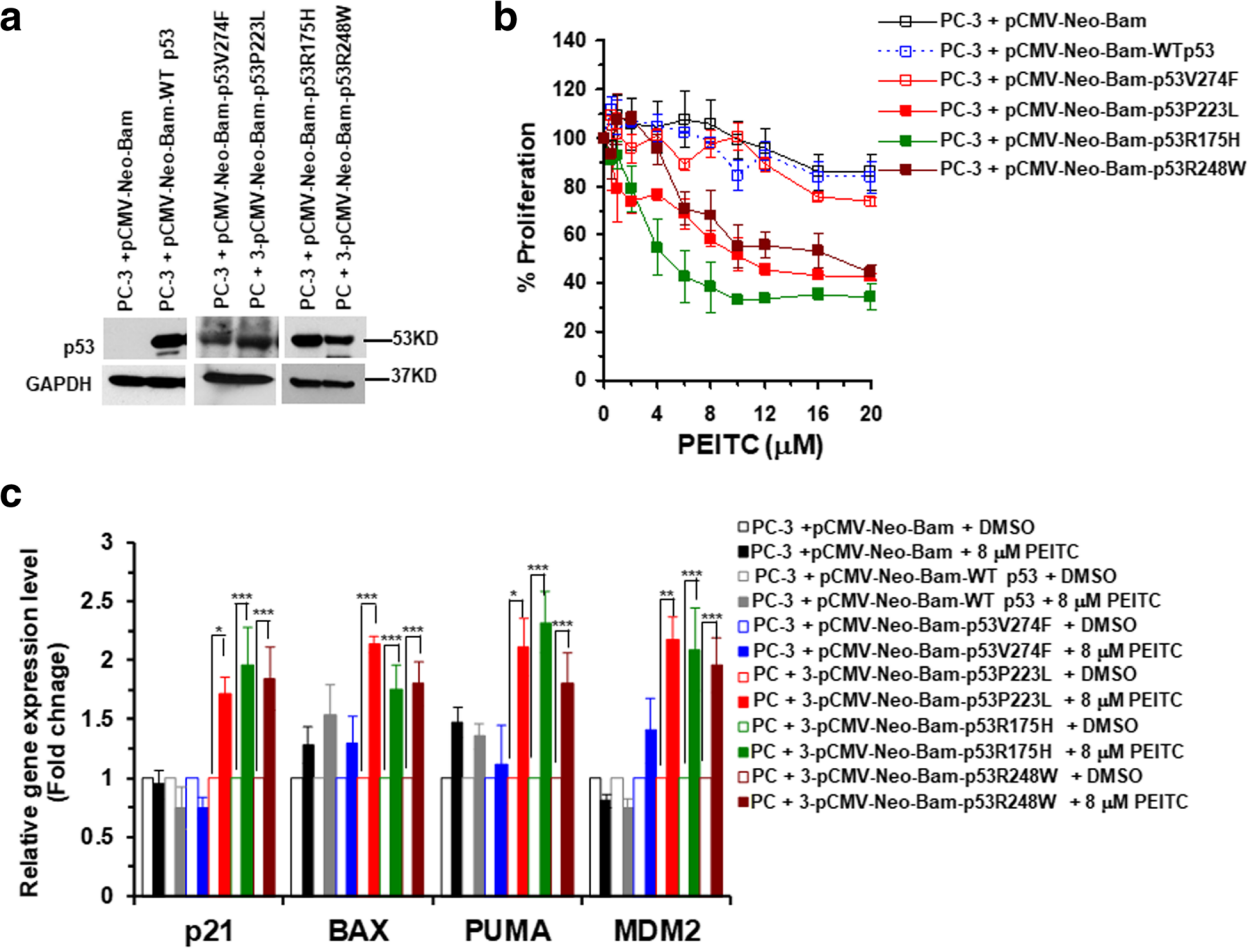
PEITC acts in a p53 mutant type-dependent manner. **a** Western blot of the isogenic PC-3 cells harboring indicated plasmids. The cell lysates were resolved by SDS-PAGE, probed with p53 DO-1 antibody and reprobed with anti-GAPDH antibody. **b** Effects of PEITC on proliferation of PC-3 cells transfected with indicated plasmids after 24 h. Percent cell proliferation was determined by the WST-1 assay. **c** qRT-PCR of p53 regulated genes in PC-3 transfected cells treated with DMSO or 8 μM PEITC for 4 h. (***p ≤ 0.0000, **p ≤ 0.001 and *p ≤ 0.02)

The DU145 prostate cancer cell co-expresses two different p53 mutants, p53^P223L^ (structural mutant) and p53^V274F^ (contact mutant). Because PEITC induced conformation change and restored transactivation functions to p53 mutant in DU145 cells, we assessed its effects on each of the p53 mutant alleles independently. For this, we transfected the human PC-3 (p53 null) prostate cancer cells with plasmid pCMV-Neo-Bam, pCMV-Neo-Bam-WT p53, pCMV-Neo-Bam-p53P223L or pCMV-Neo-Bam-p53V274F, confirmed the expression of p53 mutants in these isogenic cells by western blotting (Fig. 4a) and then treated the cells with PEITC. The pCMV-Neo-Bam-p53P223L (structural mutant) cells displayed the maximum inhibition of cell proliferation by PEITC as compared to the cells transfected with pCMV-Neo-Bam, pCMV-Neo-Bam-WT p53 or pCMV-Neo-Bam-p53V274F (contact mutant) (Fig. 4b).

We also transfected PC-3 cells with pCMV-Neo-Bam-p53R175H (structural mutant) or pCMV-Neo-Bam-p53R248W (contact mutant), confirmed the expression of p53 mutants in these isogenic cells by western blotting (Fig. 4a) and then treated the cells with PEITC. The pCMV-Neo-Bam-p53R175H and pCMV-Neo-Bam-p53R248W cells displayed the significant inhibition of cell proliferation by PEITC as compared to the cells transfected with pCMV-Neo-Bam or pCMV-Neo-Bam-WT p53 (Fig. 4b). Collectively, these results demonstrate a functional involvement of the mutational status of p53 in cells response to PEITC. Consistent with this idea, p53 knockdown in p53^R175H^ LAPC-4 cells resulted in significantly reduced sensitivity to PEITC induced growth inhibition, whereas, cells transfected with NS siRNA remained highly sensitive (Additional file 3: Figure S3a and b).

To determine the restoration of the transactivational functions to p53 mutant in PC-3-pCMV-Neo-Bam-p53R175H, PC3-pCMV-Neo-Bam-p53P223L and PC3-pCMV-Neo-Bam-p53R248W cells, we evaluated the mRNA levels of p53 canonical target genes p21, BAX, MDM2 and PUMA in these cells treated with PEITC. As controls mRNA levels of p53 regulated downstream target genes were evaluated after PEITC treatment in PC-3 cells transfected with plasmid pCMV-Neo-Bam, pCMV-Neo-Bam-WTp53 or pCMV-Neo-Bam-p53V274F. PEITC (8 μM) induced a significant increase in the expression of the WT p53 canonical targets in PC-3 cells harboring structural (p53^R175H^ or p53^P223L^) or contact (p53^R248W^) mutant compared to the cells harboring vector only, WT p53 or p53^V274F^ mutant (Fig. 4c). These results demonstrate that PEITC restored transactivation functions in DU145, LAPC-4 and VCaP cells are caused by reactivation of p53^P223L^, p53^R175H^ and p53^R248W^ mutants, respectively, and, importantly, PEITC act in a p53 mutant type-dependent manner.

### PEITC induces a delay in cell cycle progression and activates ataxia telangiectasia mutated (ATM) in DU145 and LAPC-4 cells

**Fig. 5:**
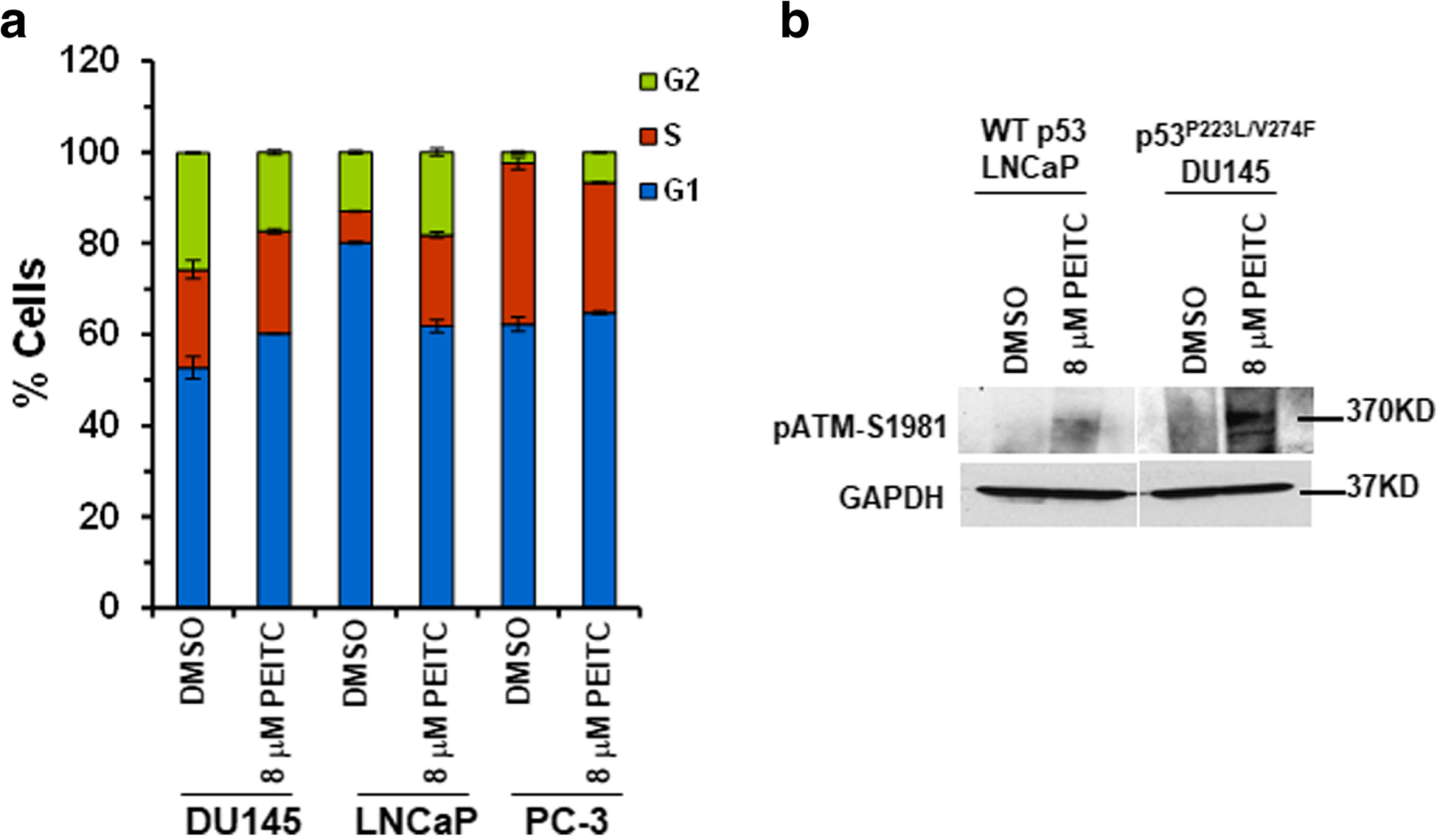
PEITC induces G1 phase arrest and activates ATM. **a** Cells were treated with DMSO or 8 μM PEITC for 24 h and analyzed by flow cytometry. **b** DU145 and LNCaP cells were treated with DMSO or 8 μM PEITC for 4 h. Blots were probed using anti-pATM S1981 antibody. As a control blots were probed with anti-GAPDH antibody

WT p53 plays a crucial role in regulating cell cycle progression and in maintaining genetic stability. PEITC restored transactivation functions to p53 mutant in DU145 cells, therefore, we examined if reactivated p53 mutant was able to modulate cell cycle progression. DU145 cells treated with PEITC (8 μM) displayed a significant increase in G1 phase population (60% compared with 52.7% in DMSO-treated control) (Fig. 5a), suggesting that PEITC inhibited cell proliferation by delaying cells in G1 phase. The WT p53 LNCaP cells treated with PEITC (8 μM) showed an increase in S and G2/M- phase at 24 h (Fig. 5a). The p53 null PC-3 cells treated with PEITC (8 μM) did not show any significant change in cell cycle progression (Fig. 5a). These results further support that PEITC restores “WT” like functions to p53 mutant in DU145 cells.

PEITC induces accumulation of reactive oxygen species (ROS), exerts oxidative stress and elevates DNA double strand breaks (DSBs) [15, 10, 27]. DNA DSB damage activates ATM pathway in WT p53 cells [28]. One of the important GOF activities of p53 mutant is its ability to impair activation of ATM after DNA DSB damage by inhibiting its recruitment to the sites of DNA damage and induces genetic instability [29, 30]. Therefore, we examined if PEITC induced restoration of mutant p53 in DU145 cells abolishes it ability to inhibit activation of ATM under conditions of DNA damage accumulation. Cells were treated with 8 μM PEITC or DMSO and western blot analysis was performed to detect activation of ATM. As a control LNCaP cells were treated similarly. Western blot analysis showed autophosphorylation of ATM at S1981 in DU145 cells treated with PEITC, whereas no significant phosphorylation was detected in DMSO control (Fig. 5b). These results suggest that the absence of inhibition of ATM by p53 mutant leads to the reactivation of the DNA damage response. We also detected a mild accumulation of pATM-S1981 in LNCaP cells treated with 8 μM PEITC consistent with the cell- cycle data as shown previously (Fig. 5a).

Next, we examined if PEITC induced restoration of p53 mutant affects cell cycle progression and activates ATM in p53^R175H^ LAPC-4 cells. LAPC-4 cells treated with 8 μM PEITC displayed a significant increase in G1 (40% compared with 30.4% in DMSO-treated control) and S (33% compared with 17.2% in DMSO-treated control) phase populations (Additional file 4: Figure S4a), suggesting that PEITC inhibited cell proliferation by delaying cells in G1 and S phase at 24 h. Consistent with the delay in cell cycle progression, we detected a significant accumulation of auto-phosphorylated ATM at S1981 in LAPC-4 upon PEITC treatment (Additional file 4: Figure S4b). Collectively, these results suggest that PEITC induced restoration of the transactivation functions to p53 mutant and reactivation of the DNA damage response leads to apoptosis in p53^P223L/V274F^ DU145 and p53^R175H^ LAPC-4 cells.

### Inhibition of growth by PEITC occurs in a p73-independent manner

p73, a member of the p53 family, is a transcription factor that shares significantly high structural and functional homology, including the DNA binding domain, with p53 [31–33]. As a GOF activity, mutant p53 interacts with and sequesters p73, thus, inhibiting its transcriptional activities [34–36]. To determine if PEITC induced restoration of mutant p53 affected it ability to interact with p73, we measured the mRNA levels of p73 in PEITC treated p53^R175H^ LAPC-4, p53^R248W^ VCaP and p53^P223L/V274F^ DU145 cells. As a control we measured mRNA levels of p73 in PEITC treated WT p53 LNCaP cells. PEITC (8 μM) enhanced the mRNA levels of p73 in LAPC-4, VCaP and DU145 cells, expressing different p53 mutant-types (Additional file 5: Figure S5). We also detected an increase in the mRNA levels of p73 in PEITC treated LNCaP cells (Additional file 5: Figure S5). These results suggest that p53 mutant-rescue by PEITC resulted in the release of the sequestered p73.

Next, we investigated if p73 plays a role in PEITC induced inhibition of growth and induction of apoptosis in p53 mutant prostate cancer cells. We used DU145 cells that co-express a structural and a contact p53 mutant. We treated DU145 cells transfected with plasmid HA-p73α-pcDNA3 with PEITC. As a control, DU145 cells transfected with plasmid pcDNA3 were treated similarly. The DU145 cells overexpressing p73 did not display any significant increase in the inhibition of cell proliferation and induction of apoptosis by PEITC as compared to the DU145 cells transfected with plasmid alone (Additional file 6: Figure S6a and b). Furthermore, PEITC treatment did not induce p73 expression levels in p53 mutant DU145 cells (Additional file 6: Figure S6c). To further confirm that p73 was not involved in PEITC induced- p53 pathway activation, we measured the mRNA levels of p53 target gene p21 in PEITC treated DU145 cells transfected with plasmid HA-p73α-pcDNA3. The p73 over-expression in DU145 cells did not result in any significant increase in expression of p21 compared to the DU145 cells transfected with vector alone (Additional file 6: Figure S6d). These results demonstrate that p73 is not involved in PEITC induced inhibition of cell growth and apoptosis induction in mutant p53 DU145 cells.

### PEITC inhibits growth and reduces the expression levels of mutant p53 in DU145 xenograft tumor

**Fig. 6:**
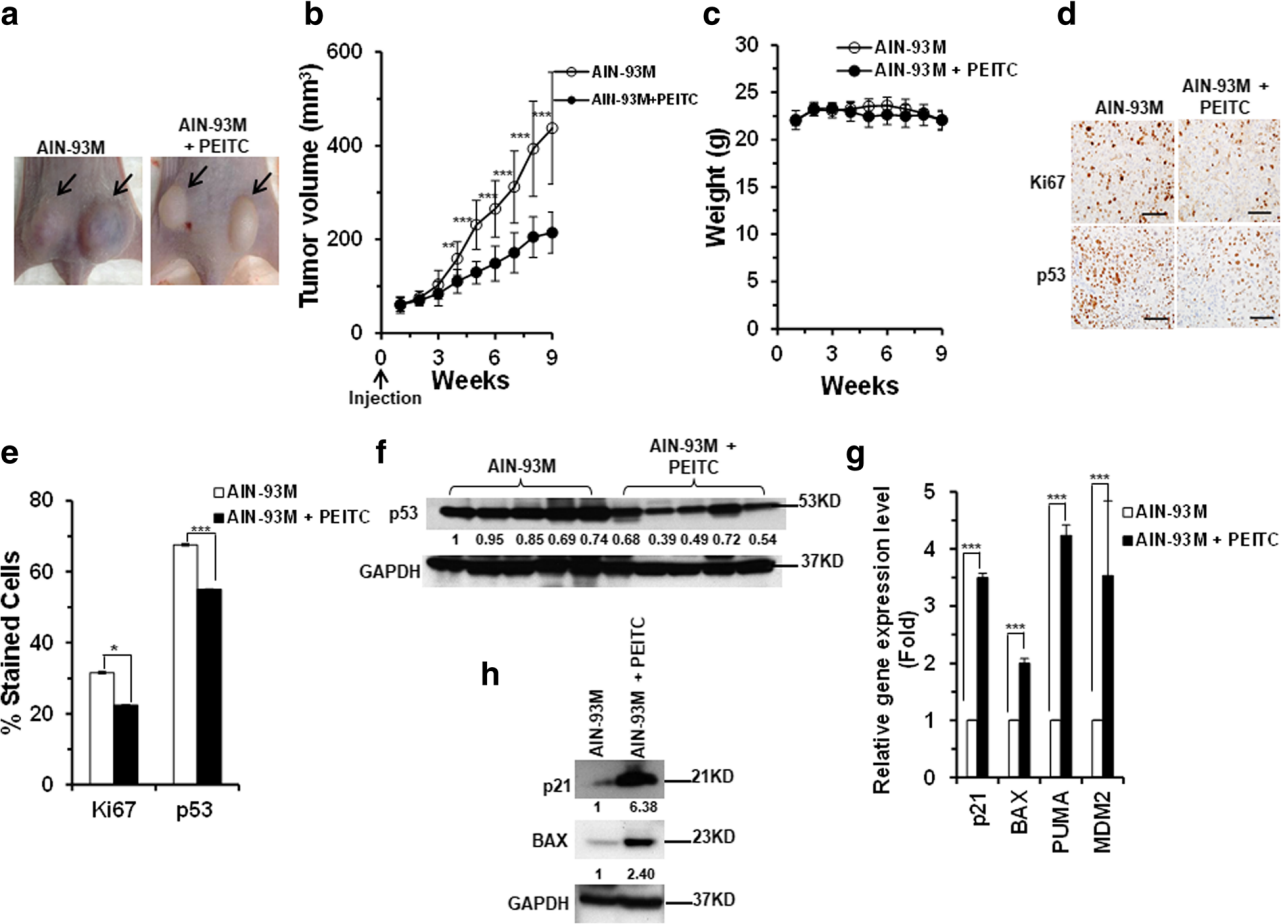
In vivo evidence of dietary-PEITC mediated p53^P223L/V274F^ mutant reactivation and growth inhibition of DU145 tumors. **a** Representative images of DU145 prostate xenograft tumors in control (Left Panel) and PEITC-treated (Right Panel) animal groups. **b** Tumors volumes in animals. (***p ≤ 0.0001 and **p ≤ 0.003) (*n* = 14). **c** Animal weights (g) were measured weekly. **d** Representative images of tumor tissue stained for Ki67 and p53. All scale bars represents a size of 200 μm. **e** Percentage of Ki67 (*p ≤ 0.01) and p53 (***p ≤ 0.00005) positive cells in tumor tissue sections (*n* = 10). Results are expressed as ± SD. **f** Western blot analysis of p53 expression levels in the tumors from control and PEITC-treated animal groups. Blot shown is representative of the 12 tumor tissue lysates analyzed from each group. **g** qRT-PCR (*n* = 4) of p53 regulated genes in PEITC and control animal groups. Results are expressed as ± SD. (***p ≤ 0.0003). **h** Western blot of p21 and Bax expression in DU145 xenograft tumors in vivo

The growth inhibitory potential of PEITC in DU145 cells and its ability to reactivate p53 mutant prompted us to evaluate its effects on DU145 xenograft tumor in vivo. Male athymic nu/nu Balb/c mice (CAnN.Cg-Foxn1nu/Crl, 4–6-week old) was fed a PEITC diet (5 μmol/ g AIN-93 M diet) a week before the injection of 2 × 10^6^ DU145 cells into the right and left flanks of the animals (“cancer chemoprevention” settings). The animals in the control group were given an AIN-93 M diet. Tumor formation was assessed and tumor volumes were measured during the 9 weeks bioassay period. A statistically significant inhibition of tumor growth (> 50%, ***p ≤ .0001 and **p ≤ 0.003) was observed in mice on the PEITC diet as compared to those on the control diet (Fig. 6a and b). No difference in body weights was observed between the groups (Fig. 6c). Further, the histological examination of the tumor tissue sections revealed a statistically significant reduction in the expression of proliferation antigen Ki67 in tumors from mice on PEITC diet (Fig. 6d and e). These results demonstrate that PEITC has anti-tumor activity in the human prostate DU145 xenograft model.

Because PEITC induced a significant reduction in the expression levels of mutant p53 in DU145 in vitro, we assessed its effects on p53 mutant protein in vivo. We examined the levels of mutant p53 protein in tumor tissue sections and lysates by immunohistochemistry (IHC) and immunoblotting, respectively. Compared to the tumors in the control group, tumors in the PEITC group showed a statistically significant decrease in the percentage of p53 mutant stained cells (Fig. 6d and e). Furthermore, a significant reduction of p53 protein mutant expression levels was detected in animals on PEITC diet (Fig. 6f). The variability in the reduced levels of p53 mutant in the PEITC treated mice may be because of the inherent differences in this animal model. However, the mutant p53 levels were consistently lower in the PEITC treated group than the control group. Together, these results demonstrate that PEITC can deplete mutant p53 protein in vivo.

### PEITC induces the restoration of the transactivational functions to p53 mutant in human prostate DU145 xenograft tumor in nude mice

We assessed whether PEITC induces p53 mutant reactivation in vivo by qRT-PCR and immunoblot analysis of xenograft tumor tissue lysates from PEITC treated and control groups. A significant elevation in mRNA levels of canonical p53 target genes p21, BAX, PUMA and MDM2 was detected in animals fed PEITC diet as compared to the animal in the control group (Fig. 6g). Further, immunoblot analysis revealed a significant increase in p21 and BAX proteins in the tumor tissue lysates of animals fed PEITC diet (Fig. 6h). Together, these results provide evidence that PEITC can reactivate p53 mutant in vivo and inhibits DU145, that co-express two different type of p53 mutants, prostate xenograft tumor growth in mouse model.

### p53 mutant-rescue by PEITC inhibits its ability to interact with p73 in vivo

We determined whether PEITC induced restoration of p53 mutant affects its ability to interact with p73 in vivo by qRT-PCR analysis of p53 ^P223L/V274F^ DU145 xenograft tumor tissue lysates from PEITC treated and control groups. A significant elevation in mRNA levels of p73 gene was detected in animals fed PEITC diet as compared to the animals in the control group (Additional file 7: Figure S7). Further, increased expression of p73 was also detected in the p53^R175H^ LAPC-4 xenograft tumor tissue lysates of animals fed PEITC diet (Additional file 7: Figure S7). Together, these results provide evidence that p53 mutant-rescue by PEITC can inhibit its GOF activity in vivo. Importantly, the inhibition occurred in xenograft tumors expressing different p53 mutants.

## Discussion

Mutations in the p53 gene occur in a variety of human cancers with remarkably high frequencies (as high as 95%), depending upon the type and stage of the tumors [37]. The majority (~ 70%) of mutations are missense that are localized to six “hotspot” residues, which play a role either in the structural integrity (structural mutant, e.g. R175, P223) or in the DNA binding domain (contact mutant, e.g. R248, R273) [38, 39]. These mutations lead to loss of contact with consensus DNA binding sequence, thus resulting in loss of WT p53 tumor suppressive activity. Importantly, the p53 mutants can also exert a “dominant negative” effect on WT p53 activity or acquire new tumorigenic (GOF) activities rendering the mutants oncogenic [40–43]. Humans with Li-Fraumeni syndrome, an autosomal-dominant disorder due to germline mutations in p53 gene, are predisposed to tumorigeneisis [44]. Therefore, targeting mutant p53 offers a potentially promising approach for cancer prevention and therapy. However, studies investigating the role of mutant p53 as a target for dietary-related cancer chemopreventive compounds are scarce.

PEITC, a compound derived from watercress, possesses cancer chemopreventive potential in various animal models, and epidemiological studies also lend support to the role of dietary ITCs in the protection against certain human cancers [1–3]. Furthermore, PEITC has been studied in clinical phase 1 and phase 2 trials (http://www.clinicaltrials.gov/ct2/results?term=PEITC). Although, several activities have been proposed for PEITC [1–13], our observation that PEITC selectively depletes mutant p53, and not WT p53 [14], has led us to conduct an in-depth investigation of the underlying mechanism. Recently, we discovered a novel mechanism of PEITC that it reactivates a hotspot p53^R175H^ structural mutant in SK-BR-3 breast cancer cells in vitro and in vivo resulting in tumor inhibition [15]. The diversity of p53 mutations in human cancers demands a better understanding of the effects of PEITC on different cancer types and p53 mutants.

In this study, we showed that PEITC inhibited the growth of prostate cancers expressing different hotspot p53 mutants, p53^R175H^ and p53^R248W^, with differential potencies, by restoring transactivation functions to both mutants, suggesting that PEITC acts in mutation type-dependent manner. In addition, our data revealed that by affecting p53 mutant stability, PEITC also affects the sensitivity of the p53 protein to degradation pathways [15]. Taken together, PEITC-induced reactivation of p53 mutant(s) and the subsequent decrease in the expression levels of mutant protein(s) likely constitute the underlying mechanism for its anti-cancer activities.

Reinforcing this idea that PEITC can reactivate different p53 mutant(s), we showed that PEITC inhibited proliferation and induced apoptosis in DU145 cells, the human prostate cancer cells co-expressing a structural (p53^P223L^) and a contact (p53^V274F^) mutant. PEITC restored WT p53 transactivation functions to p53 mutant in DU145 cells. Studies with isogenic p53 null PC-3 cells expressing each of the two p53 mutants independently revealed that PEITC induces significant inhibition of cell proliferation and selectively restores the transactivation functions to mutant p53 in PC-3 cells expressing p53^P223L^ structural mutant, but not p53^V274F^ contact mutant. Further mechanistic studies are needed to understand the reason for the selectivity of PEITC towards P223L mutant. Furthermore, expression of p53^R175H^ (structural mutant) or p53^R248W^ (contact mutant) significantly enhanced the sensitivity of the isogenic PC-3 cells to PEITC compared to the PC-3 cells transfected with vector alone or WT p53. Collectively, these results suggest that PEITC acts in a p53 mutant type-dependent manner and preferentially towards structural mutants.

PEITC-caused oxidative stress can lead to the accumulation of DNA DSBs that activates the restored p53 mutant and induces apoptosis [15]. We found PEITC treatment induced phosphorylation of ATM in p53 mutant p53^P223L/V274F^ DU145 and p53^R175H^ LAPC-4 cells, suggesting that reactivation of the DNA damage response might be responsible for the activation of the restored p53 mutant and induction of apoptosis. In support of this, we observed that PEITC induces G1 phase delay and G1 and S phase delay in DU145 and LAPC-4 cells, respectively. These results also demonstrate that PEITC induced p53 mutant-rescue abolishes its GOF activity to inhibit ATM activation caused by DSB damage in different p53 mutants.

The p73 tumor suppressor inhibits cell-proliferation or induces apoptosis in response to DNA damage and other stress stimuli that activate p53 [31–33]. Mutant p53 binds with and sequesters p73 inhibiting its transcription activities [34–36]. We showed that restoration of p53 mutant by PEITC inhibits its ability to interact with p73 as evidenced by increased mRNA levels of p73. We also investigated the possible role for p73 in PEITC-induced p53-pathway activation. We showed that the effects of PEITC in DU145 cells are p73-independent. Consistent with this, PEITC did not induce expression of p73 protein or mRNA levels of p21 in DU145 cells transfected with HA-p73α-pcDNA3 compared to DU145 cells transfected with vector only, further supporting the role of reactivated p53 mutant in the inhibition of growth and apoptotic induction in DU145 cells by PEITC. Collectively, these results suggest that PEITC-induced restoration of mutant p53 also inhibits its GOF activities. It would be of interest to further study the effects of PEITC on other GOF activities of the restored mutant p53, including its ability to bind to and modulate activities of transcription factors or proteins [30, 45, 46].

Our animal bioassay, designed as a “chemoprevention study”, demonstrated that, like the in vitro data, p53 mutant can be targeted in vivo by PEITC. Animals were fed a PEITC containing diet a week before the injection of p53^R175H^ mutant LAPC-4 or p53^P223L/V274F^ mutant DU145 cells. The cells injected into the right and left flanks of the mice mimic the presence of “initiated” or “transformed” cells that have potential to form tumors. Since the animals lack the palpable tumors initially, our mouse model is distinct from previously described xenograft models under “chemotherapeutic settings” where drugs were administered directly into the palpable tumors [47, 48]. PEITC resulted in a statistically significant reduction (> 50%, *p* < 0.05) in tumor volumes. The elevated mRNA levels of canonical p53 target genes in prostate cancer LAPC-4 cells provide proof-of-concept that dietary PEITC can reactivate p53^R175H^ mutant in a different cancer cell type. Consistent with our in vitro results, PEITC reactivates mutant p53 in vivo in prostate cancer DU145 cells that expresses two different p53 mutants, p53^P223L^ structural and p53^V274F^ contact, resulting in tumor inhibition. These results demonstrate that PEITC can reactivate different structural p53 mutants in vivo.

Consistent with our previous findings of preferential inhibition by PEITC of cells expressing p53^R175H^ mutant (the third most common missense mutation in human cancers, with an estimated 5.1% frequency of alteration [38, 49]) in breast (SK-BR-3, AU565) (IC_50_ 4 and 6 μM, respectively) and lung (HOP-92) (IC_50_ 2 μM) [15], here we demonstrated that among the cells examined, p53^R175H^ expressing LAPC-4 prostate cancer cells (IC_50_ 4 μM) are the most sensitive to PEITC induced growth inhibition. In p53 null PC-3 isogenic cell lines, the pCMV-Neo-Bam-p53R175H (structural mutant) cells displayed significantly higher sensitivity to PEITC as compared to the cells transfected with pCMV-Neo-Bam-p53P223L (structural mutant) or pCMV-Neo-Bam-p53R248W (contact mutant). These results suggest that the observed differences in the sensitivity of prostate cancer cells to PEITC was p53 mutant-type specific. Furthermore, VCaP cells expressing p53^R248W^ mutant displayed an IC_50_ (12 μM) that is similar to p53^R248Q^ OVCAR3 (IC_50_ 12 μM) cells shown previously [15]. Collectively these studies suggest that PEITC-induced inhibition of tumor growth is likely to be independent of cancer organ-site of origin. Furthermore, PEITC can reactivate different p53 mutants; structural or contact; however, more studies are required to establish the mutation-type dependent nature and broad action applicability of PEITC. Importantly, these results support the potential of PEITC in “basket trials” for cancers harboring selective p53 mutations irrespective of their site of origin. The finding that PEITC induced restoration of mutant p53 abolishes its GOF activity to inhibit activation of ATM raises the possibility to develop novel strategies in which PEITC can be used in combination with chemotherapeutic drugs that work by inducing DNA damage to target genomic instability in cancers. Consistent with this idea, we showed previously that PEITC acts synergistically with cisplatin to enhance apoptosis [50].

We propose a mechanism involving the binding of PEITC to the mutant p53 protein and its binding affinities vary depending upon the site of mutation and the conformation of the p53 mutant protein. This paradigm is consistent with the findings that different p53 mutants display different sensitivities to PEITC-induced inhibition of proliferation and apoptosis induction. In addition, our previous studies showed that PEITC induces “WT-like” conformation in purified p53^R175H^ mutant protein as well as in the mutant p53^R175H^ protein in SK-BR-3 and HOP-92 cells [15]. The present study showed that PEITC-induced conformational change in the mutant p53 protein in DU145 cells in a mutant type-specific manner. Furthermore, we showed earlier that ITCs bind the cysteine residues in purified p53 DNA binding domain and that the binding affinities seem to correlate with their ability to deplete mutant p53 in cells [14]. Consistent with this idea, we demonstrated PEITC depleted p53^P223L/V274F^, p53^R175H^ and p53^R248W^ mutant proteins to different levels. More studies are needed to fully understand the mechanism(s) of p53 mutation site-dependent reactivation by PEITC.

Recent studies on comprehensive characterization of the prostate cancer transcriptome and genome have revealed that p53 gene represents one of the most frequently mutated driver gene in primary prostate cancer [51] and mutations in p53 gene also occur at remarkably high frequency in metastatic tumors and metastatic castration-resistant prostate cancer (mCRPC) [52–54]. Mutations in the p53 gene occur at different stages of prostate tumorigenesis from early stage prostate cancer to its invasive or metastatic tumors or mCRPC, suggesting that p53 mutant is a potential target for therapeutic interventions in this disease. In principle, pharmacological reactivation of the mutant p53 presents a viable strategy to selectively target cancer cells. Synthetic small molecules that restore p53 point mutant to a transcriptionally competent form have been identified from chemical libraries [47, 48, 55–57]. Although some of the small molecules have been used in clinical trials [57], none has reached clinics, which underlines the need to discover new pharmacological alternatives, with higher selectivity and lower toxicity. Studies exploring the potential of naturally-occurring compounds of dietary origin that can target p53 mutants are rare. These studies are important to discover leads for developing practical and effective strategies for target-based cancer treatment and prevention. The presence of p53 mutants in prostatic intraepithelial neoplasia [33], a premalignant condition that gives rise to prostate cancer, suggests the potential of dietary PEITC in the prevention of this disease. The demonstration of targeting selective p53 mutant by PEITC as a critical mechanism to inhibit prostate cancer provides a rationale for implementing a gene-targeted strategy to prevent or treat these cancers.

## Conclusions

In conclusion, this is the first report that via mutant p53 reactivation PEITC, a naturally-occurring compound derived from cruciferous vegetable, kills prostate cancer cells harboring different “hotspot” p53 mutants (structural and contact) to differential extents, but preferentially toward structural mutants. We also showed that PEITC inhibits the growth of prostate xenograft tumors harboring different p53 mutants and reactivates different p53 mutants in vivo. Previously, we have shown that PEITC reactivates mutant p53 in vitro as well as in a breast cancer SK-BR-3 xenograft mouse model, thus, inhibiting tumor growth. Collectively, these results suggest that the anticancer activity of PEITC is cancer type-independent, yet it acts in a p53 mutant type-dependent manner. These findings set stage for novel and practical personalized treatment / prevention strategies for human prostate cancers with p53 mutations. The results also support the potential of PEITC as a “basket trial” agent for human cancers harboring specific p53 mutant, irrespective of the organ-site-of-origin.

## Additional files

Additional file 1: **Figure S1.** Effects of PEITC on apoptosis in LAPC-4 and VCaP prostate cancer cell lines. Representative pictures of flow cytometry data show effects of PEITC on apoptosis in LAPC-4 and VCaP cells treated with DMSO or 8 μM PEITC for 24 h as measured by Annexin-V staining using a BD LSRFORTESSA instrument. (TIF 154 kb)

Additional file 2: **Figure S2.** Effects of PEITC on apoptosis in LNCaP, DU145 and PC-3 prostate cancer cell lines. Representative pictures of flow cytometry data show effects of PEITC on apoptosis in DU145, LNCaP and PC-3 cells treated with DMSO or 8 μM PEITC for 24 h as measured by Annexin-V staining using a BD LSRFORTESSA instrument. (TIF 221 kb)

Additional file 3: **Figure S3.** Effects of PEITC on proliferation of p53^R175H^ knockdown LAPC-4 cells. LAPC-4 cell line was transfected with non specific (NS) siRNA or p53 siRNA as described in Materials and Methods. (a) Effect of p53 siRNA on p53 expression level in LAPC-4 cells was then determined by western blot analysis. Thirty μg of the cell lysate was resolved by SDS-PAGE and probed with anti-p53 DO-1 antibody. Blot was stripped and reprobed with anti-GAPDH antibody. (b) LAPC-4 cell line transfected with NS siRNA or p53 siRNA was treated with DMSO (control) or the indicated concentrations of PEITC for 24 h. Percent cell proliferation was determined by the WST-1 assay. (TIF 70 kb)

Additional file 4: **Figure S4.** PEITC delays cell cycle progression and activates ATM in p53^R175H^ LAPC-4 cells. (a) LAPC-4 cells were treated with DMSO or 8 μM PEITC for 24 h and cell cycle progression was analyzed by flow cytometry. (b) LAPC-4 cells were treated with DMSO or 8 μM PEITC for 4 h. Blot was probed using anti-pATM S1981 antibody. As a loading control blot was probed with anti-GAPDH antibody. (TIF 128 kb)

Additional file 5: **Figure S5.** Effects of PEITC on mRNA levels of p73 gene in prostate cancer cell lines. qRT-PCR of p73 gene in mutant p53 (LAPC-4, VCaP, DU145) or WT p53 (LNCaP) cells treated with DMSO or 8 μM PEITC for 4 h. Results are expressed as ± SD. (***p ≤ .0000, **p ≤ 0.005 and *p ≤ 0.02). (TIF 61 kb)

Additional file 6: **Figure S6.** PEITC inhibits growth in a p73-independent manner. DU145 cells transfected with HA-p73α-pcDNA3 or pcDNA3 were treated with PEITC for 24 h. (a) Percent cell proliferation was determined by the WST-1 assay, and (b) Apoptosis was measured by Annexin-V staining by flow cytometry using a BD LSRFORTESSA instrument. Left Panel; representative pictures of flow cytometry data, Right Panel; quantification of the data. (***p ≤ 0.0000 and *p ≤ 0.02). (c) DU145 cells transfected with HA-p73α-pcDNA3 were treated with DMSO or the indicated concentration of PEITC for 4 h. Blots were probed with anti-p73 and anti-p53 (p53 DO-1) antibodies and reprobed with anti-GAPDH antibody. (d) qRT-PCR of p21 gene in DU145 cells transfected with HA-p73α-pcDNA3 or pcDNA3 and treated with PEITC for 4 h. Results are expressed as ± SD. (***p ≤ 0.0000 and **p ≤ 0.002). (TIF 602 kb)

Additional file 7: **Figure S7.** Effects of PEITC on mRNA levels of p73 gene in p53^R175H^ LAPC-4 and p53^P223L/V274F^ DU145 xenograft tumors. qRT-PCR (*n*=5) of p73 gene in xenograft tumor tissues of animals in PEITC and control diet fed groups. Results are expressed as ± SD. (***p ≤ 0.000). (TIF 53 kb)

## Data Availability

Data sharing is not applicable to this article as no datasets were generated or analysed during the current study.

## Abbreviations

ATM: Ataxia telangiectasia mutated
DSBs: Double strand breaks
FBS: Fetal bovine serum
IHC: Immunohistochemistry
ITCs: Isothiocyanates
PEITC: Phenethyl isothiocyanate
qRT-PCR: quantitative RT-PCR
ROS: Reactive oxygen species
WT: Wild-type
