# Supplementary figures and images for: p53 mutant-type in human prostate cancer cells determines the sensitivity to phenethyl isothiocyanate induced growth inhibition
Source: J Exp Clin Cancer Res. 2019 Jul 15;38:307. doi: 10.1186/s13046-019-1267-z (PMC6632191; doi:10.1186/s13046-019-1267-z)

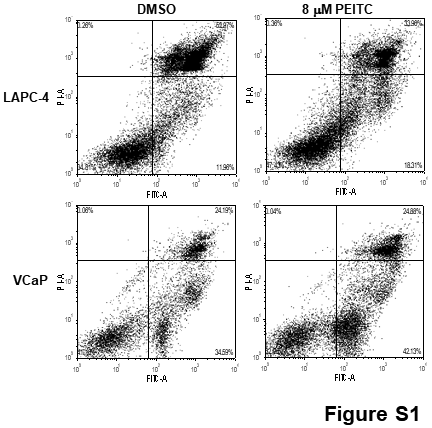

Supplement: Supplementary file 1 — Figure S1. Effects of PEITC on apoptosis in LAPC-4 and VCaP prostate cancer cell lines. Representative pictures of flow cytometry data show effects of PEITC on apoptosis in LAPC-4 and VCaP cells treated with DMSO or 8 μM PEITC for 24 h as measured by Annexin-V staining using a BD LSRFORTESSA instrument. (TIF 154 kb) [file 13046_2019_1267_MOESM1_ESM.tif]

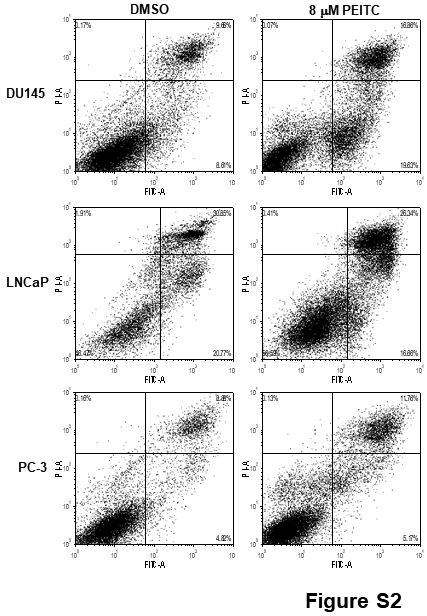

Supplement: Supplementary file 2 — Figure S2. Effects of PEITC on apoptosis in LNCaP, DU145 and PC-3 prostate cancer cell lines. Representative pictures of flow cytometry data show effects of PEITC on apoptosis in DU145, LNCaP and PC-3 cells treated with DMSO or 8 μM PEITC for 24 h as measured by Annexin-V staining using a BD LSRFORTESSA instrument. (TIF 221 kb) [file 13046_2019_1267_MOESM2_ESM.tif]

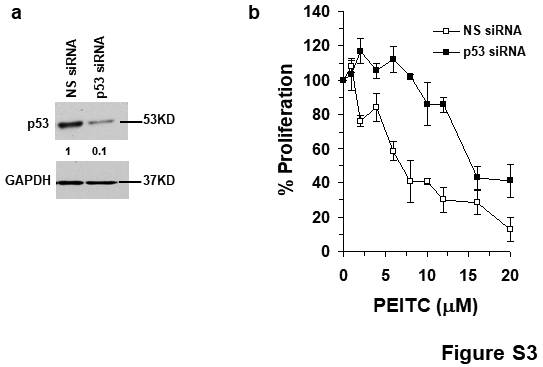

Supplement: Supplementary file 3 — Figure S3. Effects of PEITC on proliferation of p53R175H knockdown LAPC-4 cells. LAPC-4 cell line was transfected with non specific (NS) siRNA or p53 siRNA as described in Materials and Methods. (a) Effect of p53 siRNA on p53 expression level in LAPC-4 cells was then determined by western blot analysis. Thirty μg of the cell lysate was resolved by SDS-PAGE and probed with anti-p53 DO-1 antibody. Blot was stripped and reprobed with anti-GAPDH antibody. (b) LAPC-4 cell line transfected with NS siRNA or p53 siRNA was treated with DMSO (control) or the indicated concentrations of PEITC for 24 h. Percent cell proliferation was determined by the WST-1 assay. (TIF 70 kb) [file 13046_2019_1267_MOESM3_ESM.tif]

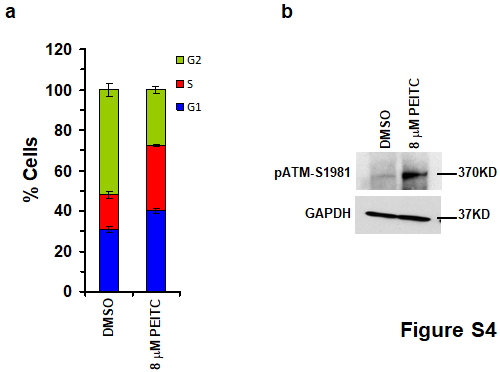

Supplement: Supplementary file 4 — Figure S4. PEITC delays cell cycle progression and activates ATM in p53R175H LAPC-4 cells. (a) LAPC-4 cells were treated with DMSO or 8 μM PEITC for 24 h and cell cycle progression was analyzed by flow cytometry. (b) LAPC-4 cells were treated with DMSO or 8 μM PEITC for 4 h. Blot was probed using anti-pATM S1981 antibody. As a loading control blot was probed with anti-GAPDH antibody. (TIF 128 kb) [file 13046_2019_1267_MOESM4_ESM.tif]

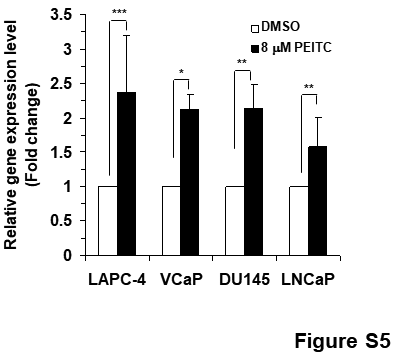

Supplement: Supplementary file 5 — Figure S5. Effects of PEITC on mRNA levels of p73 gene in prostate cancer cell lines. qRT-PCR of p73 gene in mutant p53 (LAPC-4, VCaP, DU145) or WT p53 (LNCaP) cells treated with DMSO or 8 μM PEITC for 4 h. Results are expressed as ± SD. (***p ≤ .0000, **p ≤ 0.005 and *p ≤ 0.02). (TIF 61 kb) [file 13046_2019_1267_MOESM5_ESM.tif]

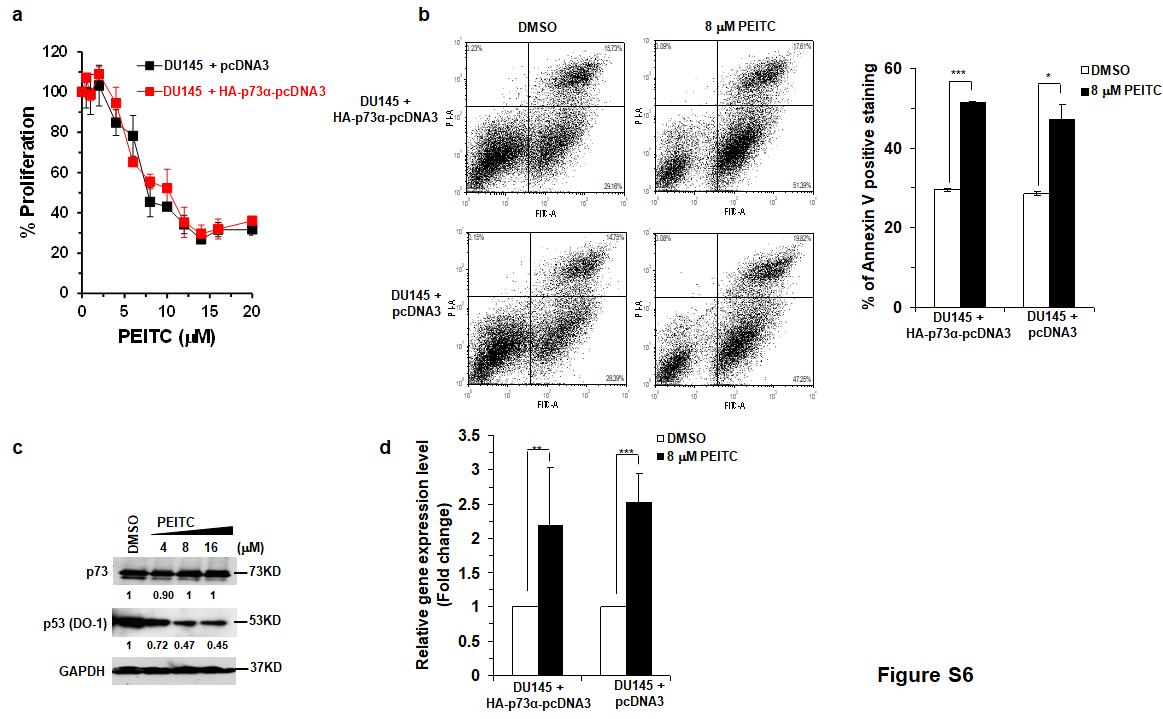

Supplement: Supplementary file 6 — Figure S6. PEITC inhibits growth in a p73-independent manner. DU145 cells transfected with HA-p73α-pcDNA3 or pcDNA3 were treated with PEITC for 24 h. (a) Percent cell proliferation was determined by the WST-1 assay, and (b) Apoptosis was measured by Annexin-V staining by flow cytometry using a BD LSRFORTESSA instrument. Left Panel; representative pictures of flow cytometry data, Right Panel; quantification of the data. (***p ≤ 0.0000 and *p ≤ 0.02). (c) DU145 cells transfected with HA-p73α-pcDNA3 were treated with DMSO or the indicated concentration of PEITC for 4 h. Blots were probed with anti-p73 and anti-p53 (p53 DO-1) antibodies and reprobed with anti-GAPDH antibody. (d) qRT-PCR of p21 gene in DU145 cells transfected with HA-p73α-pcDNA3 or pcDNA3 and treated with PEITC for 4 h. Results are expressed as ± SD. (***p ≤ 0.0000 and **p ≤ 0.002). (TIF 602 kb) [file 13046_2019_1267_MOESM6_ESM.tif]

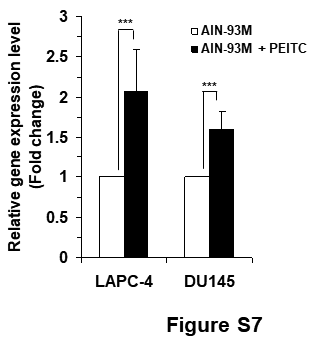

Supplement: Supplementary file 7 — Figure S7. Effects of PEITC on mRNA levels of p73 gene in p53R175H LAPC-4 and p53P223L/V274F DU145 xenograft tumors. qRT-PCR (n=5) of p73 gene in xenograft tumor tissues of animals in PEITC and control diet fed groups. Results are expressed as ± SD. (***p ≤ 0.000). (TIF 53 kb) [file 13046_2019_1267_MOESM7_ESM.tif]
